# Spine radiosurgery for the local treatment of spine metastases: Intensity-modulated radiotherapy, image guidance, clinical aspects and future directions

**DOI:** 10.6061/clinics/2016(02)09

**Published:** 2016-02

**Authors:** Fabio Ynoe de Moraes, Neil Kanth Taunk, Ilya Laufer, Wellington Furtado Pimenta Neves-Junior, Samir Abdallah Hanna, Heloisa de Andrade Carvalho, Yoshiya Yamada

**Affiliations:** I Hospital Sírio-Libanês, Departamento de Radioterapia, São Paulo/, SP, Brasil; II Hospital das Clínicas da Faculdade de Medicina da Universidade de São Paulo, Instituto de Radiologia, Serviço de Radioterapia, São Paulo/SP, Brasil; III Memorial Sloan Kettering Cancer Center, Department of Radiation Oncology, New York/NY, USA; IV Memorial Sloan Kettering Cancer Center, Department of Neurosurgery, New York/NY, USA

**Keywords:** Stereotactic Body Radiotherapy, SBRT, Spine Radiosurgery, Spine Tumors, Spine Metastasis, Image-Guided Radiotherapy, Clinical Trial

## Abstract

Many cancer patients will develop spinal metastases. Local control is important for preventing neurologic compromise and to relieve pain. Stereotactic body radiotherapy or spinal radiosurgery is a new radiation therapy technique for spinal metastasis that can deliver a high dose of radiation to a tumor while minimizing the radiation delivered to healthy, neighboring tissues. This treatment is based on intensity-modulated radiotherapy, image guidance and rigid immobilization. Spinal radiosurgery is an increasingly utilized treatment method that improves local control and pain relief after delivering ablative doses of radiation. Here, we present a review highlighting the use of spinal radiosurgery for the treatment of metastatic tumors of the spine. The data used in the review were collected from both published studies and ongoing trials. We found that spinal radiosurgery is safe and provides excellent tumor control (up to 94% local control) and pain relief (up to 96%), independent of histology. Extensive data regarding clinical outcomes are available; however, this information has primarily been generated from retrospective and nonrandomized prospective series. Currently, two randomized trials are enrolling patients to study clinical applications of fractionation schedules spinal Radiosurgery. Additionally, a phase I clinical trial is being conducted to assess the safety of concurrent stereotactic body radiotherapy and ipilimumab for spinal metastases. Clinical trials to refine clinical indications and dose fractionation are ongoing. The concomitant use of targeted agents may produce better outcomes in the future.

## INTRODUCTION

The spine is the most common site of bony metastatic disease. At least 40% of patients with advanced cancer will have spinal involvement during the course of their disease and approximately 5-10% of these patients will develop some type of epidural cord compression. More than 90% of metastatic lesions involving the spine are extradural; intradural and intramedullary lesions represent <5% and <1% of lesions, respectively 1,2,3.

Up to 50% of metastatic lesions originate from breast, lung or prostate cancers. With improved systemic treatment and augmented survival times, a greater number of patients with other tumor histologies will develop secondary lesions 1.

Early diagnosis of spinal metastatic disease is critical because functional outcomes depend on neurologic condition at the time of presentation. Tumor-related pain typically appears early in the morning or at night, generally improves with physical activity and frequently precedes the development of other neurological symptoms by weeks or months 4,5.

Uncontrolled spinal tumors typically produce pain and diminish ambulatory ability and performance status. Treatment is primarily palliative and is achieved by gaining local control of the disease. In selected cases, chemotherapy or surgery may pose as alternatives. The proper management of patients with spinal metastasis requires multidisciplinary treatment managed by orthopedic surgeons, neurosurgeons, radiation oncologists, medical oncologists, pain medicine specialists, radiologists and palliative care professionals.

Although palliative radiotherapy plays a prominent role in treating patients with metastatic spine disease, there is increasing literature that supports the use of stereotactic body radiotherapy (SBRT) or spine radiosurgery (SR)-based ablative treatment 6,7,8.

SR is a recently developed radiation therapy technique that delivers high-dose radiation to a tumor while minimizing the radiation delivered to healthy, neighboring tissues. A treatment target is defined by high-resolution imaging, which is also used for treatment planning and dose calculation. Precise delineation of the spinal cord requires registration if computer tomography (CT) images to the T2-weighted magnetic resonance imaging (MRI) series, or a CT myelogram. Treatment is delivered using a combination of externally placed strict immobilization devices and image-guided (IG) intensity-modulated radiation therapy (IMRT) 9,10. The treatment course is reduced to only a few sessions (generally up to 5), with total doses ranging from 12 to 30 Gy delivered in 1 to 5 fractions.

SR aims to improve existing rates of clinical response and tumor control, reduce re-treatment rates for metastatic lesions by increasing the biologic equivalent dose^1^, minimize radiation doses to healthy organs and allow safer reirradiation in previously treated sites 11. SR spares the spinal cord better than conventional radiotherapy and fractionated IMRT, even when very high doses are prescribed to an area only a few millimeters in size. Immobilization devices and image-guidance (IGRT) tools, such as kilovoltage cone-beam imaging 12, have reduced treatment errors associated with traditional radiotherapy and enabled safe and accurate delivery of the highly conformal dose distributions produced by IMRT techniques 13.

The aim of this article was to review published data on and ongoing trials of SR for metastatic tumors of the spine.

### Review Methodology

A literature search was conducted using the MEDLINE database [via PubMed] and the Clinicaltrials.gov database for all article types available through September 2014. No other filter was activated and we assessed all of the published data that were available in English. Controlled vocabulary was leveraged as well as text words to develop the search strategy detailed below.

Our search terms and strategy used the following MEDLINE MeSH terms: ((Spinal Neoplasms [MeSH] OR Neoplasm, Spinal OR Neoplasms, Spinal OR Spinal Neoplasm OR Spine [MeSH] Vertebral Column OR Column, Vertebral OR Columns, Vertebral OR Vertebral Columns OR Spinal Column OR Column, Spinal OR Columns, Spinal OR Spinal Columns OR Vertebra OR Vertebrae) AND (Radiosurgery [MeSH] OR Radiosurgeries OR Radiosurgery, Stereotactic OR Radiosurgeries, Stereotactic OR Stereotactic Radiosurgeries OR Stereotactic Radiosurgery OR Gamma Knife Radiosurgery OR Gamma Knife Radiosurgeries OR Radiosurgeries, Gamma Knife OR Radiosurgery, Gamma Knife OR Stereotactic Body Radiotherapy OR Body Radiotherapies, Stereotactic OR Body Radiotherapy, Stereotactic OR Radiotherapies, Stereotactic Body OR Radiotherapy, Stereotactic Body OR Stereotactic Body Radiotherapies OR CyberKnife Radiosurgery OR CyberKnife Radiosurgeries OR Radiosurgeries, CyberKnife OR Radiosurgery, CyberKnife OR Radiosurgery, Linear Accelerator OR Linear Accelerator Radiosurgeries OR Radiosurgeries, Linear Accelerator OR Linear Accelerator Radiosurgery OR Radiosurgery, Linac OR Radiosurgeries, Linac OR LINAC Radiosurgery OR Radiosurgeries, LINAC)). For the Clinicaltrials.gov database, we used the following search terms: Spinal Neoplasms OR Spine AND Radiosurgery OR Radiation Therapy.

Two authors independently screened all identified studies by title and abstract. Studies with the following inclusion criteria were collected: primary focus on clinical outcome, evaluation of feasibility, assessment of toxicity and presentation of technical aspects. Additional treatment with chemotherapy, targeted therapy, endocrine therapy and surgery were permitted if radiotherapy was the primary intervention under investigation. All references in each included study were manually searched for important missed publications. Re-reporting and clinical trials were manually removed, and only the most recent publications were evaluated 14,15,16.

Studies that did not focus on SR were removed. Articles published in languages other than English were excluded. We also excluded articles with a main focus of epidemiology, research design, diagnosis, basic science or clinical guidelines. Editorial commentaries were removed. Finally, we removed studies with fewer than 20 patients, heterogeneous treatment design (multiple levels of SR dose or SR not the main objective of analysis) and less than 6 months of follow-up. A third evaluator resolved any disagreements.

### Review Results

**Figure 1-:**
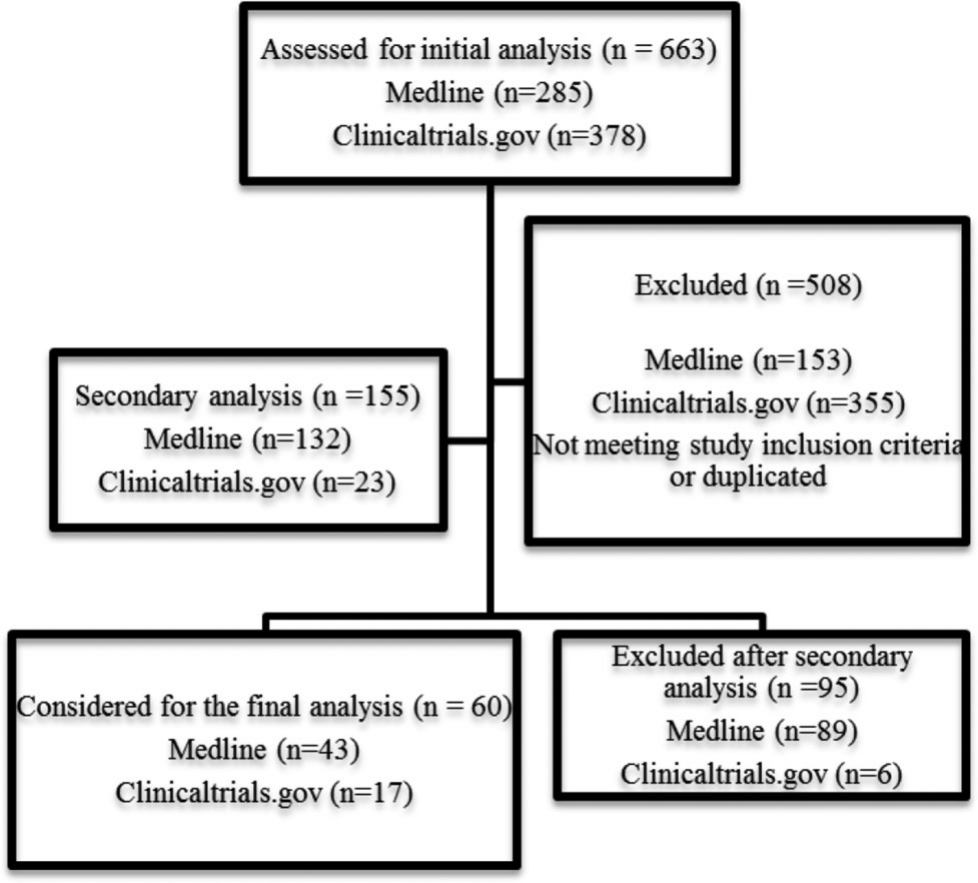
Flow diagram.

We identified 663 studies [MEDLINE n=285; Clinicaltrials.gov n=378]). After assessing and removing duplicated records (n=10), 653 studies were screened; of these, only 155 met the eligibility criteria for full-text assessment. After manually screening the full-text articles of the remaining studies (n=155 [MEDLINE n=132; Clinicaltrials.gov n=23) for our inclusion criteria, we identified 60 unique articles for qualitative analysis (Figure 1).

### Data Extraction

One investigator (F.Y.M.) independently extracted data from all 60 studies. The following data were collected: study design, year of publication, non-radiotherapy interventions (e.g., hormone therapy, chemotherapy, targeted agents or procedures), type of radiotherapy treatment course (IMRT, IGRT, proton therapy, and brachytherapy), and primary assessed outcome [overall survival (OS), local control, pain control, quality of life measures and toxicity].

### Technical Aspects of Spinal Radiosurgery

#### Dose Delivery Technique: Intensity-Modulated Radiotherapy

The use of IMRT is required to deliver high-dose radiation to vertebra while simultaneously sparing the spinal cord and other adjacent critical organs (e.g., the esophagus or brachial plexus). The development of IMRT was a major improvement over 3D conformal radiotherapy (3DCRT) 17. IMRT is capable of modulating a target-shaped field of uniform radiation intensity into hundreds of pixel-like “beamlets”, each with its own intensity. This advancement allows the delivery of increased radiation intensity to areas within the beam that are contained within the target volume only and reduces the radiation delivered to healthy organs via so-called intensity modulation. As a result, it is possible to create complex dose distributions characterized by steep dose gradients (i.e., significant dose differences over a short distance) to create a target (e.g., vertebra) with a concave shape to avoid hitting organs at risk (e.g., the spinal cord).

IMRT fields are often created through inverse planning optimization algorithms, where doses are prescribed to delineated volumes and potential plan alternatives are generated with guidance from a treatment planner (e.g., a dosimetrist or medical physicist) until a reasonable solution is found. Delivery via these intensity-modulated beams is achieved through the use of physical compensators (which are outdated technology) or, preferably, multi-leaf collimators (MLCs). An MLC serves as an add-on to a conventional rectangular linear accelerator's collimator. MLCs consist of sets of opposed leaf pairs (typically approximately 60 to 80 pairs of tungsten metal bars) that automatically longitudinally displace in and out of a beam's rectangular aperture to produce irregular custom shapes. Via the continuous or stepwise movement of the leaves during radiation emission, it is possible to modulate the intensity across a beam. This modulation of beam intensity improves the therapeutic index by allowing for increased target dose and reducing damage to normal tissues.

**Figure 2-:**
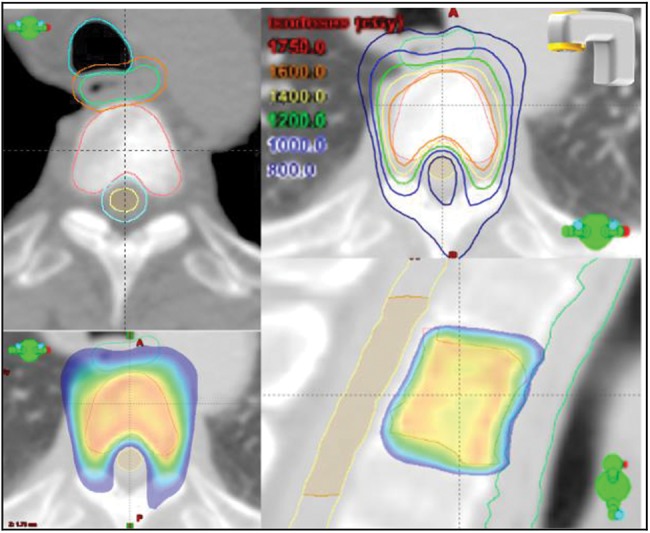
Spine radiosurgery. Target definition (vertebral body), radiotherapy isodose lines (upper right) and dose distribution (below). The axial and sagittal views illustrate “dose-shaping” promoted by IMRT, with sparing of the spinal cord and esophagus. [Male, 62 years old, metastatic melanoma of the spine, stable lesion, ECOG 0, treated with SR via 18 Gy delivered in a single fraction. The distance between the GTV (gross target volume) and the medulla is approximately 2 mm].

With the use of IMRT, a sharp dose gradient of approximately 10% per millimeter can be achieved near the spinal cord 18. For example, in a photon-based IMRT plan, where the spinal cord is 3 mm from the edge of the target volume, 18 Gy can be delivered to more than 90% of the target volume, while the maximum dose to the spinal cord surface would be less than 14 Gy (Figure 2).

**Figure 3-:**
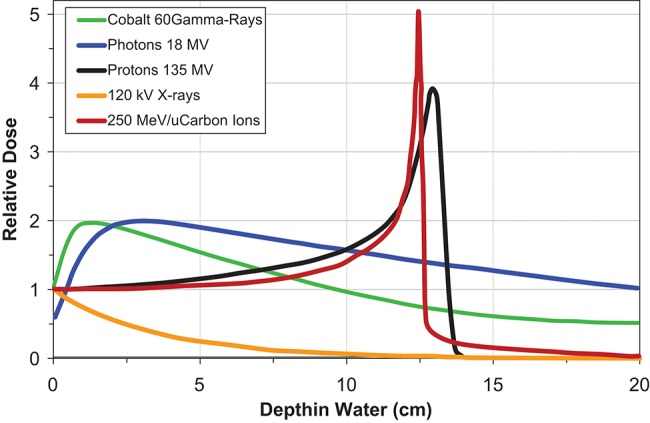
Depth dose curves for photons, cobalt 60, protons and carbon ions.

The rationale for using proton and other particle beams is the ability to achieve excellent dose distribution at a tumor target with virtually no exit dose delivered beyond the target volume. The Bragg peak phenomenon associated with particle beam radiation results in an extremely steep dose fall-off over a course of a few millimeters (Figure 3). The advantage of this characteristic lies primarily in the lack of exit dose beyond the Bragg peak. However, there is currently no routine indication to use proton radiation for SR.

#### Patient Setup: Immobilization and Image Guidance

The general treatment parameters for SR are currently based on the use of IMRT and, more recently, volumetric arc therapy (VMAT, which is dosimetrically equivalent but more efficient and faster than IMRT). In addition, SR requires the use of image guidance and setup verification. The overall precision of different components (e.g., mechanical aspects, image guidance and beam targeting) should allow treatment accuracy within 1 mm 19. The primary role of IGRT is to guide patient re-positioning based on a target itself (in this case, the vertebral body being treated). Secondly, depending on the IGRT technique, patient position can be monitored during treatment to assure that the target remains static through frequent intra-fraction imaging. IGRT also requires that a patient remain immobilized in the correct position throughout dose delivery; devices exist specifically for this purpose.

For SR, a patient is immobilized in a stereotactic body frame or immobilization cradle. Such devices are noninvasive and thus do not guarantee that a patient will remain perfectly positioned. Nonetheless, based on pre- and post-treatment imaging findings, an approximate 95% rate of immobilization accuracy has been reported for the noninvasive devices that are currently in use 20. Immobilization has emerged as an issue of great importance because planning technology is ineffective unless a patient is accurately positioned.

Accurate positioning is possible through patient cooperation. After proper positioning is achieved, image acquisition, ideally using onboard cone-beam imaging just before RT treatment, can be initiated. Pretreatment images are registered and compared with target images acquired during simulation to identify and correct setup errors. Typically, these variations are minimal and account for displacements of less than 3 millimeters from the ideal position. These displacements are accounted for by repositioning the patient to match the target before treatment begins. After corrections are made in each direction, spine positioning can be considered truly reliable for a treatment session, with less than 1 mm of error 21.

#### Clinical Assessment and Patient Selection

Due to the improved OS gained by improving local and systemic therapies and the capacity of SR to improve local tumor control, SR has great potential for increased utilization. Spine metastatic disease is often observed in radiation oncology clinics and studies of SR have indicated that the technique results in excellent local control rates 8,22,23. The indications for SR are still expanding; they currently include pain related to an involved vertebral body, radiographic tumor progression, lesions associated with progressive neurologic deficits, adjuvant therapy after surgical intervention for radioresistant metastasis and the need for reirradiation 8.

Memorial Sloan Kettering Cancer Center (MSKCC) utilizes a multidisciplinary spine team to identify and treat patients who would most benefit from SR. The MSKCC Spine Clinic developed and now implements the algorithm NOMS for metastatic spine disease. This algorithm incorporates assessments of (N)eurologic condition, (O)ncologic status, (M)echanical instability and (S)ystemic disease 24. The goal of this decision framework is to provide a rapid, highly reliable assessment (e.g., low-grade extradural spinal cord compression (ESCC) *versus* high-grade disease or mechanical *versus* biologic pain) for the treatment of spinal metastases. Such treatment integrates radiation therapy, interventional neuroradiology and surgical approaches (Table 1) 24,25.

#### Local Disease Control

Local control is defined as the absence of recurrent cord compression within an irradiated field or the absence of progression at a treatment site. The local control achieved with SR has been assessed in many series. Several of these series are highlighted below.

The Radiation Therapy Oncology Group (RTOG) published a phase II study evaluating the use of SR for localized spinal metastases (RTOG 0631). In this multicenter phase II study, patients with 1-3 lesions and a numerical rating pain scale score of ≥5 were treated. A total of 44 patients received 16-18 Gy of single-fraction SR. Grade 1/2 and grade 3 SBRT-related adverse events were observed in seven and zero patients, respectively. The investigators concluded that SR is safe and feasible in the multi-institutional setting 10. Several departments have also published their experiences, which are summarized in Table 2 22,32,45,.

For metastatic spinal tumors, SR has been postoperatively utilized in several series and in cases of progression following conventional fractionated palliative radiation treatment. Even in these challenging settings, SR achieves local control in approximately 85-94% of cases 22,23,26,18,27. When used as a primary treatment, long-term radiographic tumor control has been demonstrated in 90% of cases 22,.

^In an assessment of 103 consecutive spinal metastases in 93 patients without high-grade ESCC who were treated with SBRT (median dose, 24 Gy delivered in a single fraction; range, 18-24 Gy), MSKCC reported an overall local control rate of 90% at a median follow-up of 15 months. There were no deaths attributed to local failure. No patient experienced myelopathy or radiculopathy, even when the maximum spinal dose was raised to 14 Gy from 12 Gy ^^23^.

In another phase I/II trial, 149 patients (166 metastatic spine lesions) were treated with SR. None of the patients showed evidence of mechanical instability or cord compression and 50% of them exhibited radioresistant tumor histology. Each of the patients received a total dose of 27-30 Gy, typically delivered in three fractions. More than half of the patients had been treated with one prior course of conventional radiation therapy (30 Gy delivered in ten fractions). The local tumor progression-free survival reported after SR was 81% at 1 year and 72% at 2 years. Adverse events were minimal and SR was associated with robust pain reduction (26% of the patients were pain-free before SR and 54% were pain-free 6 months after SR). SR also reduced pain medication use and was associated with improved quality of life 31.

In another report, 88 patients with 120 spinal metastases from pathologically proven high-grade sarcoma were treated with hypofractionated [3-6 fractions; median dose, 28.5 Gy; n=52 (43.3%)] or single-fraction SR [median dose, 24 Gy; n=68 (56.7%)]. At a median follow-up of 12.3 months, the 12-month local control rate was 87.9%, and the OS was 60%. Single-fraction SR showed better local control than hypofractionation (*p*=0.007). The rate of adverse effects was low, and no grade >3 toxicity was reported 32.

#### Pain Control

Despite the use of heterogeneous methods for pain evaluation (e.g., verbal analysis scale and subjective measurements of pain relief, among others), most series report very high pain control rates (up to 96%) at 12 months 8. Pain control rates were still high even in cases of radioresistant tumors, such as melanoma, sarcoma, renal cell tumors, non-small cell lung cancer, gastrointestinal tumors and others (96% and 85% rates of pain control were achieved for melanoma and renal cell carcinoma, respectively) 8,30,33. SR has also been reported to provide better pain control more rapidly than conventional radiotherapy, with pain relief sometimes observed within 24 hours of treatment 34.

It is thought that conventional radiation doses may be sub-therapeutic for radioresistant tumors. A systematic review of randomized controlled trials (RCTs) reported that conventional radiotherapy can successfully palliate bone pain in 50 to 80% of patients, but complete pain relief is only achieved in up to 30% of patients 35,36. Thus, SR is feasible for the treatment of spinal metastases and promotes high rates of local control and pain relief with low rates of toxicity.

#### Postoperative Irradiation

It has been suggested that patients with metastatic spinal cord compression (MSCC) will benefit more from high-dose and/or SR radiotherapy than they would from low-dose fractioned radiotherapy. Surgery is fundamental the management of patients with symptomatic MSCC or high-grade spine compression. Patchell et al. reported in a RCT that surgical decompression is significantly beneficial. A larger number of patients who underwent surgery plus postoperative RT regained the ability to walk compared to those who received only RT. Ambulatory status was also maintained for longer in the surgery plus RT arm 37.

However, the exact criteria for surgery in cases of MSCC remain controversial. The following factors are usually necessary for improved outcomes: favorable PS and expected OS, a relatively radioresistant tumor type and MSCC accompanied with mechanical instability 24,25,38,39. Epidural disease is an important limiting factor with respect to SR efficacy. Al-Omair et al. reported that epidural disease progression is the most common cause of treatment failure after SR and a significant predictor of poor local control 40.

To more safely administer an ablative radiation dose, it may be possible to perform separation surgery to create a space of 2–3 mm between a metastatic lesion and the spinal cord. This allows a full dose to be administered to the entire tumor volume while minimizing the radiation dose delivered to the spinal cord. The surgical procedure is performed using epidural decompression and spinal stabilization without gross total or en bloc tumor resection 41,42.

Recently, MSKCC has published their experiences with 186 patients treated from 2002 to 2011 who were managed with separation surgery followed by SR (hypofractionated or single fraction) 43,44. In this cohort, 136 patients exhibited high-grade cord compression or MSCC and the full cohort received spinal decompression surgery followed by SR within a median of 48 days after surgery. Postoperative SR provided durable local control, with a cumulative local progression incidence of 16.4% at 1 year (95% confidence interval: 10.7–22.2).

Thus, for patients presenting with spinal cord compression, separation surgery followed by SR is a safe and effective treatment option. Integrative treatment (i.e., SR and separation surgery) may reduce the extent of surgery and also provide faster and more effective radiotherapy treatment.

#### Reirradiation of Spinal Metastases

SR is an indicated treatment for cases requiring reirradiation of spinal levels and may be associated with superior tumor control rates and pain relief relative to conventional radiotherapy. However, care must be taken when a reirradiation volume either contains or is very close to the spinal cord, esophagus or other organs at risk. In the reirradiation setting, SR provides a local control rate of 66-93% and a low rate of toxicity when treatment constraints and quality assurances are respected 45,46,47.

#### Complications

A pain flare or acute worsening of pain has been found to occur in 20% of cases after SR. Such a flare may occur within 24 hours of single-dose radiation or a few days after the use of a hypofractionated scheme 48,49. The pain is usually transient and can be managed with corticosteroids or non-steroidal anti-inflammatory drugs. Radiation-induced spinal cord injury can be a severe event, but it is not often observed. The best method of preventing myelopathy is avoidance of unnecessary radiation to the spinal cord and adherence to planned dose constraints.

Vertebral fracture (VCF) is a common event after SR. VCF has an incidence of approximately 20% and is associated with higher doses per fraction (≥20 Gy) and with 3 of the 6 original Spinal Instability Neoplastic Score (SINS) 50 components, including baseline VCF, a lytic tumor and spinal deformity 51. Attention must be paid to identifying high-risk patients who would benefit from prophylactic kyphoplasty.

Other events, such as risk of mucositis and skin dermatitis, have also been associated with SR. Additionally, there are many factors that may influence SR toxicity. Such factors include tumor proximity to and extension in adjacent normal tissues, concurrent systemic therapy and targeted therapy and comorbidities (e.g., acute infection, prior surgery, diabetes, collagen vascular disease, or any genetic predisposition).

Thus, in SR, end-to-end patient assessment, planning and dosimetry are important for ensuring a safe treatment procedure.

#### Future Perspectives: Randomized Data and Combination Targeted Therapy

Multiple prospective and retrospective series on SR have been reported. There are increasing expectations for randomized phase III trials, prospective phase II studies and the use of molecularly targeted agents as a new approach to treatment. Data from phase I, II and III trials are summarized in Table 3 9,52,53,.

The RTOG is currently enrolling patients for a phase III trial, which is estimated to include 380 patients. The anticipated completion of primary data collection for this trial is July 2015 9. The trial compares 2 groups of patients: the first arm consists of patients undergoing SR (16 Gy delivered in a single fraction), while the second arm consists of patients undergoing conventional external beam radiotherapy treatment (800 cGy delivered in a single fraction). The patients will be stratified according to number of spine metastases treated (1 *vs*. 2-3) and radioresistant tumor classification (with radioresistant tumors including soft tissue sarcomas, melanomas, and renal cell carcinomas). The primary outcome is to determine whether SR improves pain control (measured by the 11-point Numerical Rating Pain Scale [NRPS]) compared with conventional external beam radiotherapy. The following secondary outcomes are also included: pain response and pain/lesion control at a treated site(s) compared with conventional external beam radiotherapy, as measured by the NRPS; local control; adverse events measured by NCI CTCAE v3.0 criteria; and long-term side effects (24 months) of SR on vertebral bone (e.g., vertebral compression fractures) and spinal cord, as measured by MRI.

MSKCC and collaborators are conducting a phase II RCT with an estimated enrollment of 200 patients and a primary anticipated completion date of October 2015. This trial includes patients over 18 years in age with metastatic lesions in the bone, spine, soft tissue and lymph nodes that have not been previously irradiated. The patients will be randomized into 2 groups: the first arm will receive SR using a single dose of 24 Gy and the second arm will be treated with fractioned SR using 27 Gy delivered in 3 fractions 30. The primary outcome is a comparison of loco-regional control rates at 24 months. The secondary outcomes are a toxicity comparison at 24 months, determination of failure patterns between the two cohorts at 24 months, and SUV uptake as a measure of tumor response and changes in tumor perfusion evaluated by dynamic contrast-enhanced (DCE)-MRI.

The Sidney Kimmel Comprehensive Cancer Center at Johns Hopkins University is currently conducting a phase I study that aims to assess the safety of prescribing either SR or stereotactic brain radiosurgery (SRS) in combination with ipilimumab (Bristol-Myers Squibb, New York, NY) to treat patients with newly diagnosed brain or spinal metastases from melanoma. The estimated enrollment is 30 patients, with a primary completion date of December 2016. The primary outcomes are a description of the number and severity of adverse events at 24 months and an assessment of the safety profile of SBRT with concurrent ipilimumab. The secondary outcomes include estimates of local control rates in the brain and spine at 24 months, determination of the systemic control rate and evaluation of progression-free survival 59. Recently published data have reported that using a combination of ipilimumab plus SRS for melanoma brain metastasis is well tolerated and associated with better loco-regional control and possibly better survival rates. However, there was a 20% rate of grade 3 or 4 toxicity using this treatment modality 53.

A retrospective series evaluated 106 metastatic renal cell carcinoma patients (55 spine and 51 brain metastases) who were treated with simultaneous standard sorafenib or sunitinib (anti-angiogenic therapy) and stereotactic radiosurgery (SRS) or SR. The patients received an average dose of 20 Gy per lesion (range, 19–20 Gy) 54. The study showed no skin toxicity, neurotoxicity or myelopathy augment the adverse effects of anti-angiogenic therapy. Additionally, no treatment-related deaths or late complications were reported at 15 months. The local control rates for cerebral lesions at 12 and 24 months were 100% and 96.6%, respectively; for spinal lesions, the local control rates at 12 and 24 months were 94.1% and 90.4%, respectively. This series demonstrated that the use of SR / SRS plus anti-angiogenic therapy in this setting is safe and provides excellent local control.

The use of SR for metastatic tumors of the spine is safe and offers high local control rates. There are extensive data regarding pain control and local control; however, these data are mostly derived from retrospective and nonrandomized prospective series. Further studies are needed to determine appropriate SR fractionation schedules and clinical indications. Two RCTs (clinicaltrials.gov numbers NCT00922974 and NCT01223248) are ongoing and may provide the data needed to gain better insight into the factors that constitute optimal therapy. There is increasing interest in and a subsequent need to characterize combination drug therapy with SR to enhance local control and even improve survival in select groups of patients.

## AUTHOR CONTRIBUTIONS

All authors were involved in the conception, methodology design, data collection and interpretation, writing, revision and final analysis of this manuscript.

## Figures and Tables

**Table 1:** Current NOMS decision framework for high-grade epidural spinal cord compression (ESCC) (Adapted from Laufer et al. 24).

Neurologic	Oncologic	Mechanical	Systemic	Decision
**High-grade ESCC ± myelopathy**	Radiosensitive	Stable		CRT
				Unstable		Stabilization followed by CRT
	Radioresistant	Stable	Able to tolerate surgery	Decompression / stabilization followed by SR
	Radioresistant	Stable	Unable to tolerate surgery	CRT
	Radioresistant	Unstable	Able to tolerate surgery	Decompression / stabilization followed by SR
	Radioresistant	Unstable	Unable to tolerate surgery	Stabilization followed by CRT

CRT: Conventional radiotherapy; SR: Spine radiosurgery.

**Table 2:** Important clinical results following spine radiosurgery (based on recent prospective and retrospective data).

Author	Study design	SR indication	N / Dose	IGRT	Reported results
Ahmed et al. 55	Prospective case series	SM: primary and reirradiation	66 patients(85 lesions)24 Gy (SF and HF)	BodyFix or thoracic-T double vacuum system*ExacTrac X-ray system with a 6D robotic couch^#^	mOS (12 months): 52.2%LC (1 year): 91.2%No grade 4 toxicity
Amdur et al. 56	Prospective case series	SM: primary and reirradiation	21 patients(12 reirradiation)Dose: 15 Gy	Customizedbody mold / onboard cone-beam CT	OS (12 months): 60%43% pain reliefNo grade 3 or 4 toxicity
Garg et al. 57	Prospective case series	SM: primary SR	61 patients(63 tumors)16-24Gy (SF)	BodyFix / stereotactic localizer and target frame	LC (18 months): 88%?Supports SR as first-line treatment
Klish et al. 58	Prospective case series	SM: reirradiation	58 patientsDose: variable	BodyFix / stereotactic localizer and target frame	LC (12 months): 89.3%< 5% of isolated failures of the nonirradiated adjacent vertebral body = focal SR feasible
Garg et al. 45	Prospective case series	SM: reirradiation	59 patients(63 tumors)30 Gy (5 fractions)27 Gy (3 fractions)	BodyFix stereotactic body frame system / stereotactic localizer and target frame	OS (12 months): 76%LC (12 months): 100%Freedom from neurological injury: 92%
Gerszten et al. 22	Prospective nonrandomized cohort study	SM: primary and reirradiation	500 patients12.5 - 25 Gy (SF and HF)	CyberKnife: Aquaplast facemask$	LC: 90% of primary lesions and 88% of lesions treated for radiographic tumor progressionClinical improvement (previous neurologic deficit): 84%Long-term pain improvement: 86%
Haley et al. 59	Prospective case series	Efficacy and cost effectiveness of CRT versus SR	44 patients(22 CRT and 22 SR)Dose: variable	CyberKnife system	Similar pain reliefNo late complications in either groupCRT: more acute toxicities and was more likely to require additional interventions
Folkert et al. 32	Retrospective case series	SM: primary and reirradiation (sarcoma)	120 lesions(SF and HF 24 Gy)	Noninvasive customized cradle / onboard imaging (orthogonal KV imaging and cone-beam CT)	OS 60%LC (12 months) 87.9%Low toxicity reported
Zelefsky et al. 60	Retrospective case series	SM: primary and reirradiation (renal cell)	105 lesions(SF and HF)Dose: 24 Gy	Customized cradle / digitally reconstructed radiographs from the simulation studies for each field's beam's eye view	PFS (36 months - HF): 17%PFS (36 months -SF 24 Gy): 88%SF: improve local control

**Legend:** N: number of patients; IGRT: image-guided radiotherapy; SF: single fraction; HF: hypofractionated; SM: spine metastasis; Hypo: hypofractionated; LC: local control; OS: overall survival; PFS: progression-free survival; SR: spine radiosurgery; CRT: conventional radiotherapy; CT: computed tomography.

**<?ENTCHAR ast?>:** (Medical Intelligence, Schwabmunchen, Germany);

#(Brainlab, Feldkirchen, Germany);

%(Integra- Radionics Burlington, MA);

$(Aquaplast Corp., Wyckoff, NJ).

**Table 3:** Spine radiosurgery. Interventions and primary outcome descriptions from clinical trials registered at clinicaltrials.gov.

Study / Date started	Status	Design	Condition	Intervention	Primary outcomes
NCT01654068 61 / Jul, 2012	Recruiting	Phase II	Spinal metastasis	1) 2-3 SM: 14 Gy SF2) 1 SM: 14 Gy SF	Any skeletal-related event
NCT01223248 52 / Oct, 2012	Recruiting	Phase III	Spinal metastasis	SR 24 Gy SF *vs*. SR, 27 Gy (HF)	Loco-regional control rates
NCT01290562 62 / Jun 2011	Recruiting	Phase II	Spinal metastasis	20-24 Gy SF; or 20-24 Gy (HF)No prior RT or prior RT or Post-op	Local control: image / symptoms
NCT00573872 63 / Dec 2007	Not recruiting (active)	Phase I/II	Spinal metastasis	Phase 1: 20-25 Gy (HF) / Phase 2: 9-24 Gy (SF)	TT: Safety
NCT01849510 64 / Apr 2013	Recruiting	Phase II(2 arms)	Spinal metastasis	HF: 12×3 Gy+integrated boost 12×4 Gy / CRT 10×3 Gy	Local control
NCT02167633 65 / Jul 2014	Recruiting	Controlled (2 arms)	MSCC	Decompression surgery plus CRT / SR 16 Gy SF	Ambulatory status
NCT00853528 66 / Feb 2009	Not recruiting (active)	Phase I	Spinal metastasis	Maximum tolerated dose HF SR	Dose escalation
NCT00631670 67 / Feb 2008	Completed	Controlled	Spinal metastasis	15 Gy SF / 25×2.8 Gy	Safety
NCT01525745 68 / Jan 2012	Completed	Phase II	Spinal metastasis	SR HF / CRT 10 fractions	Pain control: NPRS
NCT01826058 69 / Apr 2013	Recruiting	Phase II	MSCC	16 -24 Gy SF / 21-36 HF	Neurologic response
NCT01254903 70 / Dec 2012	Recruiting	Phase I	MSCC	18 Gy SF	Safety
NCT00922974 9 / Nov 2009	Recruiting	Phase II: completedPhase III	Spinal metastasis	SR 16 Gy SF / CRT 1×800 cGy	Pain control
NCT01752036 71 / Mar 2013	Recruiting	Phase II	Spinal metastasis	SR: 30 Gy (HF)	Safety
NCT01347307 72 / Sep 2008	Not recruiting(active)	Phase IV	Spinal metastasis	Benign: 12-16 Gy SF; 21-27 Gy HFMetastases: 14-25 Gy SF; 21-30 Gy HF	Tumor control
NCT01231061 73 / Nov 2010	Completed	Phase II	Spinal metastasis	Arm 1:SR 24 Gy HF / SR 16 Gy SF	Pain control
NCT01950195 53 / Jun 2013	Recruiting	Phase I	Spinal metastasis	SR+ipilimumab	Safety
NCT01624220 74 / Jun 2012	Recruiting	Assignment	Spinal metastasis	SR+4 gold seeds implanted	Safety

SR: spine radiosurgery; NPRS: numeric pain rating scale; TT: tomotherapy; QA: quality assurance; SF: single fraction; HF: hypofractionated; CRT: conventional radiotherapy; Post Op.: post operative; MSCC: metastatic spinal cord compression.

## Footnotes

1 The biological equivalent dose is a correlation of different radiotherapy dose schedules (total dose and fractionation) that produce similar biological effects.
